# Association between PR interval prolongation and electro-anatomical substrate in patients with atrial fibrillation

**DOI:** 10.1371/journal.pone.0206933

**Published:** 2018-11-05

**Authors:** Katja Schumacher, Petra Büttner, Nikolaos Dagres, Philipp Sommer, Borislav Dinov, Gerhard Hindricks, Andreas Bollmann, Jelena Kornej

**Affiliations:** 1 Department of Electrophysiology, Heart Center, Leipzig, Germany; 2 Institute for Medical Informatics, Statistics, and Epidemiology, University of Leipzig, Leipzig, Germany; Ziekenhuisgroep Twente, NETHERLANDS

## Abstract

**Background:**

Atrial fibrillation (AF) is the most common sustained arrhythmia in clinical routine. Left atrial (LA) electro-anatomical remodelling in AF patients indicates disease progression and is associated with poor therapeutic success. PR interval prolongation is associated with an increased risk for AF, however, the association between LA remodelling measured as low voltage areas (LVA) during catheter ablation and PR interval is unknown. The aim of this study was to investigate the association between PR interval prolongation and LVA in AF patients.

**Methods:**

We studied 103 patients (62±12 years, 59% males, 34% persistent AF) undergoing first AF catheter ablation and presenting with sinus rhythm. PR interval prolongation was defined as PR >200ms and analysed in resting ECG before intervention. LVA were determined using high-density maps and defined as <0.5 mV.

**Results:**

There were 24 patients (23%) with PR interval prolongation and 18 patients (17%) with LVA. There were significant correlations between PR prolongation with LVA, CHA_2_DS_2_-VASc score and eGFR (r^2^ = 0.230, 0.216, and 0.307, all p<0.05). PR interval prolongation (OR 3.450, p = 0.024), persistent AF (OR 5.391, p = 0.002), and LA size (OR 1.117, p = 0.018) were significant predictors for LVA, while age (OR 1.072, p = 0.005), LVA (OR 3.450 p = 0.024) and eGFR (OR 0.962, p = 0.004) were associated with PR interval prolongation.

**Conclusions:**

Beside persistent AF and LA size, PR interval prolongation might be useful for the prediction of electro-anatomical substrate in AF patients. Larger studies are needed to confirm these results.

## Introduction

Atrial fibrillation (AF) is the most common cardiac arrhythmia. It is associated with an increased risk of dementia, heart failure and thromboembolism, leading to an increased mortality [1]. Pathophysiological AF results in electrical and later structural remodelling of the atrial myocardium (inflammation, fibrosis, and atrial dilatation) [2].

The PR interval is defined as the time needed for an electrical impulse to be transmitted from the sinus node through the atrioventricular node to the Purkinje fibers, and therefore, it represents the atrioventricular conduction and possible interferences. So far, PR prolongation without structural heart disease or additional conduction disturbances has been considered as a benign occurrence [3]. However, recent studies have demonstrated an association between PR prolongation and the incidence of AF [3, 4] and also the underlying atrial remodelling processes [5]. A significant correlation between PR interval prolongation and AF recurrence after radiofrequency ablation was also shown [6].

Low-voltage areas (LVA), also known as electro-anatomical substrate, in the left atrium represent these atrial remodelling processes and are considered to play an important role in AF progression [7, 8]. LVA can be found in 10% of patients with paroxysmal AF and in 35% of patients with persistent AF. Most often, the anterior left atrium (LA), septum, the posterior wall, and the roof are involved. LVA demand for individually adapted catheter ablation lines and post-interventional management due to higher recurrence rates than in patients without LVA [8]. By performing individually tailored substrate modification, a significantly higher arrhythmia-free survival rate compared with a conventional approach can be achieved [9].

The role of cardio-renal axis had been analysed in different studies. The impact of renal dysfunction on AF occurrence [10] and AF recurrences after intervention [11, 12] had been described. However, the role of renal impairment on PR interval prolongation is understudied.

Both PR interval prolongation and electro-anatomical substrate are markers for the progression of atrial fibrosis. The aim of this study was to analyse the association between PR interval prolongation in surface ECG and the presence of low voltage areas during AF catheter ablation.

## Methods

### Study population

The study population included 103 consecutively selected AF patients undergoing first AF radiofrequency catheter ablation at the Heart Center Leipzig and presenting in sinus rhythm. Exclusion criteria were pregnancy, age <18 or >75, valvular AF, cancer, acute or systemic inflammatory diseases. The study was approved by the local Ethical Committee (Medical Faculty, University Leipzig) and patients provided written informed consent for participation. Patients were recruited from October 2015 until April 2017. PR interval prolongation was defined as PR >200ms corresponding with the clinical definition of atrioventricular block (AVB) I° and analysed in resting ECG before intervention. Paroxysmal and persistent AF were defined according to current guidelines [1]. Paroxysmal AF was defined as self-terminating within 7 days after onset. Persistent AF lasted longer than 7 days or required drugs or direct current cardioversion for termination. In all patients, transthoracic and transesophageal echocardiography were performed prior to the ablation. All class I or III antiarrhythmic medications with exception of amiodarone were discontinued for at least 5 half-lives before the AF ablation procedure.

### Catheter ablation

The electro-anatomical mapping was performed in sinus rhythm. End-point of the catheter ablation was isolation of the pulmonary veins with proof of both exit and entrance block. The electro-anatomical voltage maps of the left atrium excluding the pulmonary veins were created using multielectrode spiral catheter with interelectrode distance 2-5-2 or ablation catheter with a 3.5 mm electrode tip and contact measurement properties (SmartTouch Thermocool (Biosense), Diamond Bar, CA, USA and TactiCath, (Abbott), Saint Paul, MN, USA) as mapping catheter. Electro-anatomical mapping was performed using 3-D electro-anatomical mapping systems (Carto, Biosense Webster, Diamond Bar, CA, USA or EnSite Precision, Abbott). In both mapping systems the cut-off values for defining LVA were identical: <0.5 mV for low voltage and <0.2 mV for dense scar. Using multipolar catheters in combination with auto-annotation algorithms (AutoMap in Precision and ConfiDense in Carto 3) the point density was >1000. In “normal” maps the number of points was 150–200, allowing a distance between neighbouring points of <10 mm. Areas of interest with discrete low voltage were mapped more detailed (<5 mm point distance). Points with insufficient catheter-to-tissue contact or inside ablation lines were excluded.

At the end of the procedure, an attempt to induce AF or left atrial macro-reentry tachycardia (LAMRT) was performed using a standardised protocol (burst stimulation with 300, 250, 200 ms from coronary sinus). According to the underlying LVA and inducible LAMRT additional ablation lines were applied.

### Blood samples

Serum creatinine levels were assessed before ablation. Estimated glomerular filtration rate (eGFR) was estimated using the CKD-EPI (Chronic Kidney Disease Epidemiology Collaboration) equation: **eGFR = 141 X min(Scr/ĸ, 1)**^**α**^ **X max(Scr/ĸ, 1)**^**-1.209**^ **X 0.993Age X 1.018 [if female] X 1.159 [if black]**, where Scr is serum creatinine, ĸ is 0.7 for females and 0.9 for males, α is -0.329 for females and -0.411 for males, min indicates the minimum of Scr/ĸ or 1, and max indicates the maximum of Scr/ĸ or 1.

### Statistical analysis

Data are presented as mean with standard deviation (SD) if normally distributed or as median [interquartile range] for skewed continuous variables and as proportions for categorical variables. The differences between continuous values were assessed using an unpaired t-test, Mann–Whitney test, Kruskal-Wallis test, and a chi-square test for categorical variables. A p-value <0.05 was considered statistically significant. All analyses were performed with SPSS statistical software version 23.

## Results

### Study cohort

Clinical characteristics of the study population are presented in Table 1. There were 24 patients (23%) with PR interval prolongation and 18 patients (17%) with LVA. PR interval prolongation was associated with higher age, the presence of LVA, a decreased renal function and higher CHA_2_DS_2_VASc score. Similarly, patients with LVA had more often persistent AF, PR interval prolongation, a larger LA and a higher CHA_2_DS_2_VASc-Score than patients without LVA.

**Table 1:** Baseline characteristics of the study population.

	n = 103
**Age, years**	62 (54–72)
**Females**	42 (41%)
**Persistent AF**	35 (34%)
**LVA**	18 (17%)
**PR interval, ms**	172 (160–194)
**PR interval prolongation (≥200 ms)**	24 (23%)
**BMI, kg/m^2^**	29 (25–33)
**eGFR ml/min/1.73m^2^**	76 (66–92)
**LA, cm^3^**	25 (22–29)
**LV-EF, %**	59 (56–63)
**CHA**_**2**_**DS**_**2**_**-VASc score**	2 (1–4)

Data presented as mean (interquartile range) or n (%)

**Abbreviations**: AF–atrial fibrillation; LVA–low voltage areas; BMI–body mass index; eGFR–estimated glomerular filtration rate; LA–left atrial; LV-EF–left ventricular ejection fraction.

**Fig 1:**
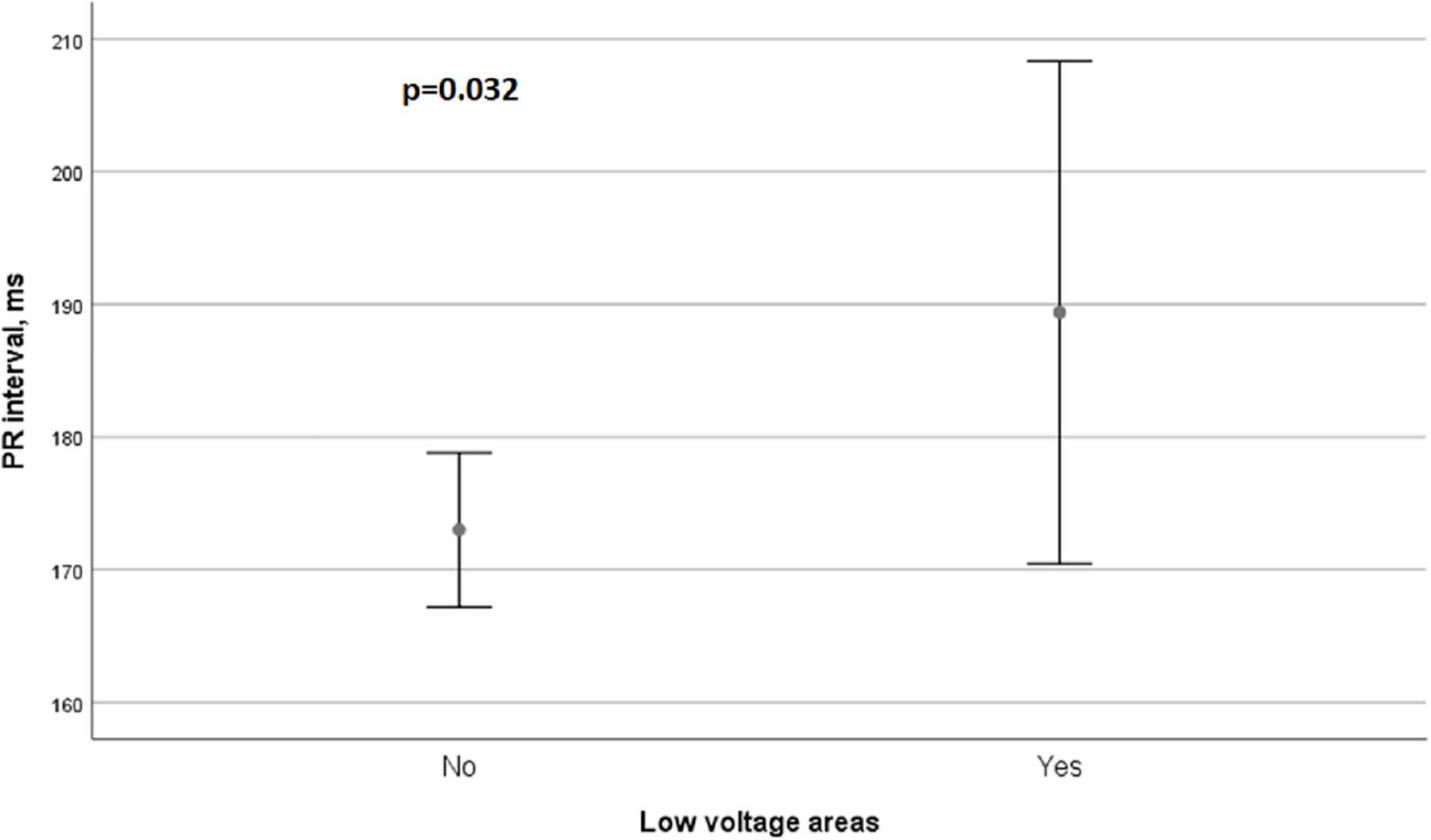
Association between PR interval prolongation and LVA.

**Fig 2:**
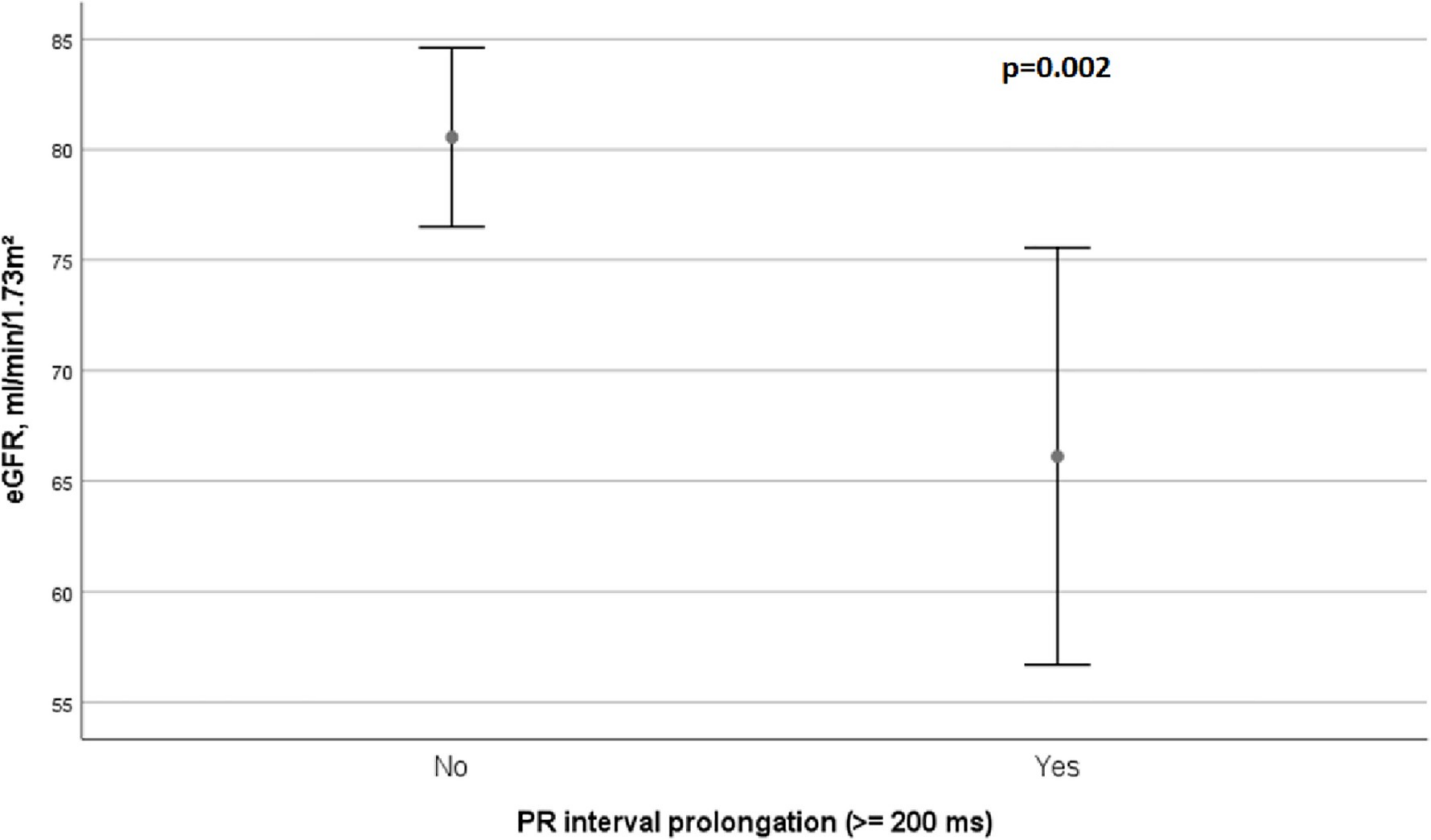
Association between PR interval prolongation and eGFR.

There were significant associations between LVA and persistent AF (r^2^ = 0.318, p = 0.001), LA size (r^2^ = 0.257, p = 0.012), CHA_2_DS_2_-VASc score (r^2^ = 0.235, p = 0.018) and PR interval prolongation (r^2^ = 0.230 p = 0.019). Patients with persistent AF had more frequently LVA (67% vs 27%, p = 0.001). Furthermore, patients with LVA had a significantly longer PR interval (189 ms vs 172 ms, p = 0.032, Fig 1). Interestingly, PR interval prolongation is attended by a decreased eGFR (p = 0.002, Fig 2).

### Associations between PR interval, LVA and clinical characteristics

On univariable analysis, age (OR 1.072, 95% CI 1.021–1.126, p = 0.005), CHA_2_DS_2_-VASc score (OR 1.363, 95% CI 1.206–1.810, p = 0.033), LVA (OR 3.450, 95% CI 1.175–10.129, p = 0.024) and eGFR (OR 0.962, 95% CI 0.889–1.004, p = 0.004) were significant predictors for PR interval prolongation (Table 2). For LVA, persistent AF (OR 5.391, 95% CI 1.812–16.044, p = 0.002), PR interval prolongation (OR 3.450, 95% CI 1.175–10.129, p = 0.024), LA diameter (OR 1.117, 95% CI 1.019–1.225, p = 0.018) and CHA_2_DS_2_-VASc (OR 1.449, 95% CI 1.056–1.990, p = 0.022) score were significant predictors, while age and gender did not reach significance (Table 2).

**Table 2:** Associations between PR interval prolongation and LVA with clinical characteristics.

	PR interval prolongation			LVA		
	OR	95% CI	p-value	OR	95% CI	p-value
**Age, years**	1.072	1.021–1.126	0.005	1.046	0.996–1.098	0.073
**Females, %**	0.836	0.327–2.140	0.709	2.737	0.962–7.787	0.059
**Persistent AF, %**	2.435	0.955–6.207	0.062	5.391	1.812–16.044	0.002
**BMI, kg/cm^2^**	0.946	0.864–1.036	0.229	1.039	0.967–1.116	0.295
**LVA, %**	3.450	1.175–10.129	0.024	-	-	-
**PR interval prolongation**	-	-	-	3.450	1.175–10.129	0.024
**LA, cm^3^**	1.076	0.995–1.170	0.066	1.117	1.019–1.225	0.018
**LV-EF, %**	0.945	0.889–1.004	0.068	0.959	0.899–1.023	0.202
**eGFR ml/min/1.73m^2^**	0.962	0.937–0.988	0.004	0.983	0.958–1.008	0.183
**CHA**_**2**_**DS**_**2**_**-VASc score**	1.363	1.026–1.810	0.033	1.449	1.056–1.990	0.022

**Abbreviations**: as in Table 1.

## Discussion

### Main findings

The main findings of our study are as follows: (1) PR interval prolongation is associated with electro-anatomical substrate assuming that PR interval could be used as a marker for atrial remodelling. (2) PR interval prolongation was associated with worse renal function. Our findings indicate that continuous monitoring of renal function in patients with PR interval prolongation might be helpful identifying patients with advanced atrial remodelling.

### PR interval prolongation—Pathophysiological aspects

The electrocardiographic PR interval reproduces the atrial and atrioventricular conduction as the time needed for an electrical impulse to be transmitted from the sinus node through the atrioventricular node to the Purkinje fibres. PR interval prolongation results from delayed impulse-conduction which may be caused by structural remodelling of atrial myocardium due to pro-fibrotic changes [2]. Several work groups found a close connection between prolonged PR interval using the clinical definition of first-degree AVB (PR >200 ms) and incidence of AF as well as other adverse cardiovascular outcomes like stroke, heart failure and dementia causing an increased mortality [3, 4, 13]. Besides the association between PR interval prolongation with onset of AF [5], higher recurrence rates after catheter ablation were described [6, 14]. However, there are also studies that could not show an association between prolonged PR interval and AF [15]. Interestingly, Nielsen et al even found a connection between short PR interval and AF [13].

### Association between LVA and PR interval prolongation

Structural remodelling in LA plays a major role in AF pathogenesis and is routinely detected invasively by LA voltage mapping during catheter ablation or non-invasively with MRI [16]. Here, electrograms with amplitudes >0.5 mV were defined as normal potentials, and signals with amplitude <0.5 mV as low-voltage potentials. Based on this connection, it is known that voltage-guided substrate modification by targeting LVA in addition to pulmonary vein isolation (PVI) is more effective than conventional PVI approaches [8, 9, 17]. Recently, Yagishita et al. showed that already a LA voltage cut-off of <1.1 mV for electro-anatomic voltage mapping in sinus rhythm can be seen as an independent predictor for recurrences in patients without LVA (<0.5 mV) [18]. Although, LVA is an important risk factor for post-procedural AF recurrences [8, 9], there is no standardised method to predict LVA non-invasively before catheter ablation procedure. In our study, PR interval prolongation was associated with advanced left atrial remodelling represented by a decrease of LA voltage. This correlation could also be shown by other research groups. Park et al. demonstrated a close association between PR interval and LA remodelling in AF patients related to higher recurrence rates after catheter ablation. Consequently, PR interval could be considered as a non-invasive predictor of clinical arrhythmia recurrence after catheter ablation [6].

### Cardio-renal axis in AF patients

The role of a cardio-renal axis had been already analysed by several work groups addressing different cardiac diseases, as heart failure [19] and arrhythmias [11]. The renal function is mainly represented by eGFR and markers of kidney damage such as proteinuria. Especially a decreased eGFR shows a strong association with AF initiation [10]. However, the data about relationship between eGFR and incident AF are inconsistent [20]. Nevertheless, lower eGFR is associated with higher recurrence rates after catheter ablation [11] and electrical cardioversion [12].

Different markers of kidney damage like proteinuria [10] and albumin excretion [20] might be associated with both AF and renal dysfunction. Also, a bidirectional relationship between AF and kidney dysfunction had been described [21]. This suggests mutual molecular pathways in both AF and renal dysfunction. In this context, Hundae et al. demonstrated that both TGF-β1 and Galectin-3 have an important function in heart as well as kidneys concerning the pathogenesis of tissue fibrosis [22]. Furthermore, there is a correlation between LA enlargement as a sign for structural remodelling reflecting a chronic exposure to hemodynamic overload due to renal disease and AF in a synergetic way [23]. Moreover, Majima et al. demonstrated an association between PR interval and eGFR decline in healthy subjects [24]. These results are in accordance with the findings in our study, where the renal dysfunction was associated with PR interval prolongation as a possible marker of atrial fibrosis found in surface ECG. Interestingly, PR interval prolongation is a predictor of increased mortality among patients with renal impairment [25, 26]. Of note, the renal function also impacts the outcome of different strategies in AF therapy [11, 12].

## Study limitations

The main limitation of the present study is a relatively small number of patients as well as small numbers of patients with PR prolongation and LVA. Therefore, our findings are hypothesis-generating and should be proven in larger prospective studies.

The impact of AV-nodal blocking medication on PR interval prolongation needs to be considered interpreting the results in this study. All patients of our cohort received AV-blocking medication as a standard medication for AF treatment, therefore, our results are consistent. Furthermore, electrolyte imbalances (especially hypo- and hyperkalemia) represent another possible interaction with PR interval. However, none of the patients from our study had significant electrolyte abnormalities.

## Conclusions

Beside persistent AF type and LA size, PR interval prolongation might be useful for the prediction of electro-anatomical substrate in AF patients. Larger studies are needed to confirm these results.
